# Tobacco Seed-Based Oral Vaccination against Verocytotoxic O138 *Escherichia coli* as Alternative Approach to Antibiotics in Weaned Piglets

**DOI:** 10.3390/antibiotics12040715

**Published:** 2023-04-06

**Authors:** Luciana Rossi, Matteo Dell’Anno, Lauretta Turin, Serena Reggi, Angela Lombardi, Giovanni Loris Alborali, Joel Filipe, Federica Riva, Pietro Riccaboni, Eugenio Scanziani, Paola Dall’Ara, Eugenio Demartini, Antonella Baldi

**Affiliations:** 1 Department of Veterinary Medicine and Animal Sciences—DIVAS, Università degli Studi di Milano, 26900 Lodi, Italy; luciana.rossi@unimi.it (L.R.); matteo.dellanno@unimi.it (M.D.); serena.reggi@unimi.it (S.R.); angela.lombardi@unimi.it (A.L.); joel.soares@unimi.it (J.F.); federica.riva@unimi.it (F.R.); pietro.riccaboni@unimi.it (P.R.); eugenio.scanziani@unimi.it (E.S.); paola.dallara@unimi.it (P.D.); eugenio.demartini@unimi.it (E.D.); antonella.baldi@unimi.it (A.B.); 2 Experimental Zooprophylactic Institute of Lombardy and Emilia Romagna (IZSLER), 25124 Brescia, Italy; giovanni.alborali@izsler.it

**Keywords:** edible vaccine, engineered plants, molecular farming, antibacterial, drug resistance, pig, post-weaning, *Escherichia coli*, toxin

## Abstract

Post-weaning diarrhoea and enterotoxaemia caused by *Escherichia coli* are serious threats in the pig (*Sus scrofa domesticus*) livestock industry and are responsible for economic losses related to mortality, morbidity and stunted growth. The aim of this study was to evaluate the effect of an engineered tobacco seeds-based edible vaccine in O138 *Escherichia coli*-challenged piglets throughout a multidisciplinary approach. Thirty-six weaned piglets were enrolled and randomly divided into two experimental groups, a control (C; *n* = 18) group and a tobacco edible vaccination group (T, *n* = 18), for 29 days of trial. At days 0, 1, 2, 5 and 14, piglets of the T group were fed with 10 g of the engineered tobacco seeds line expressing F18 and VT2eB antigens, while the C group received wild-type tobacco seeds. After 20 days, 6 piglets/group were orally challenged with the *Escherichia coli* O138 strain (creating four subgroups: UC = unchallenged control, CC = challenged control, UT = unchallenged tobacco, CT = challenged tobacco) and fed with a high protein diet for 3 consecutive days. Zootechnical, clinical, microbiological, histological and immunological parameters were assayed and registered during the 9 days of post-challenge follow up. At 29 days post-challenge, the CT group displayed a lower average of the sum of clinical scores compared to the CC group (*p* < 0.05), while the CC group showed a higher average sum of the faecal score (diarrhoea) (*p* < 0.05) than the CT group. A decreased number of days of shedding of the pathogenic strain was observed in the CT compared to the CC group (*p* < 0.05). Specific anti-F18 IgA molecules were significantly higher in the CT group compared to the CC group’s faecal samples during the post-challenge period (*p* < 0.01). In conclusion, edible vaccination with engineered tobacco seeds showed a protective effect on clinical symptoms and diarrhoea incidence during the post-challenge period, characterized by a limited time of pathogenic strain shedding in faeces.

## 1. Introduction

The weaning phase is critical for pig livestock, as it characterises the transition of piglets from lactation to self-sufficiency in feeding [1]. In particular, during the first two weeks after weaning, piglets are exposed to several stressors that could contribute to the occurrence of multifactorial diseases commonly associated with *Escherichia coli* infection [2,3]. Among *Escherichia coli* pathotypes categorized by the presence of virulence factors are verocytotoxin-producing *E. coli* (VTEC) O138, O139 and O141 strains, which cause important direct and indirect economic losses and are commonly treated with antibiotics [4,5]. In the pathogenesis, after the VTEC adhesion to the intestinal mucosa by F18 adhesive fimbriae [6,7], the verocytotoxin (VTe2) binds through the B (binding) subunits to a specific glycolipid receptor (Gb3). This leads to the internalization of the A (toxic) subunit and its subsequent translocation to the bloodstream, where it is responsible for damage to the endothelial vein cells, causing blood clots, haemorrhage, ischemic necrosis and oedema in several organs [8].

Antimicrobial resistance is currently one of the major concerns at a global level [9]. Therefore, in line with One Health principles and recent limitations on veterinary drugs (Reg. EU 6/2019), improving swine livestock sustainability is required in order to reduce and replace antibiotic treatments and re-think the farming system [10].

Generally, vaccines are considered the first alternative to antimicrobial drugs due to their preventive and specific activity against pathogens throughout the stimulation of a specific immune response [11]. Nevertheless, injectable vaccines are costly due to the need of specialized operators and impact the welfare of animals due to handling and restraint procedures [12].

In this scenario, edible vaccines could represent an interesting alternative to control *E. coli* diseases, particularly the ones caused by VTEC strains. Oral delivery of antigens has several advantages over other delivery routes, such as the ability to induce the mucosal immune response at the level of the portal of entry of the pathogen, which prevents colonization and spreading. Plant-based oral vaccines that are currently investigated, as well as having been studied during the recent COVID-19 pandemic, offer a cost-effective, needleless, convenient and safe alternative to injected vaccines [13]. In particular, seeds can be considered an interesting target for heterologous antigenic protein expression since they are natural storage organs with high protein content in a small volume, and provide a stable environment for long-term storage that does not require a cold chain [14]. Particularly, a previous study demonstrated the protective effect of individually administered tobacco seeds (mixed with chocolate and water) against a VTEC *E. coli* challenge in piglets, showing a positive impact on animal health and recovery during the post-challenge period. Moreover, the combination of multiple antigens showed a higher protective potential [12]. Therefore, the aim of this study was to evaluate, according to a previously developed multidisciplinary approach [15], the in-feed administration of tobacco seeds expressing both the VT2e-B and Fed-A subunits of F18 in piglets challenged with the O138 *E. coli* strain in order to obtain more insight on the protective effect of this vaccination strategy and the feeding protocol, and to provide data on the tobacco seed transgenic lines as a delivery system for oral vaccination.

## 2. Results

The polymorphism analysis on the FUT1 gene showed that all the piglets enrolled in the study were susceptible to F18 VTEC infection.

### 2.1. Zootechnical Performance and Clinical Scores

**Figure 1:**
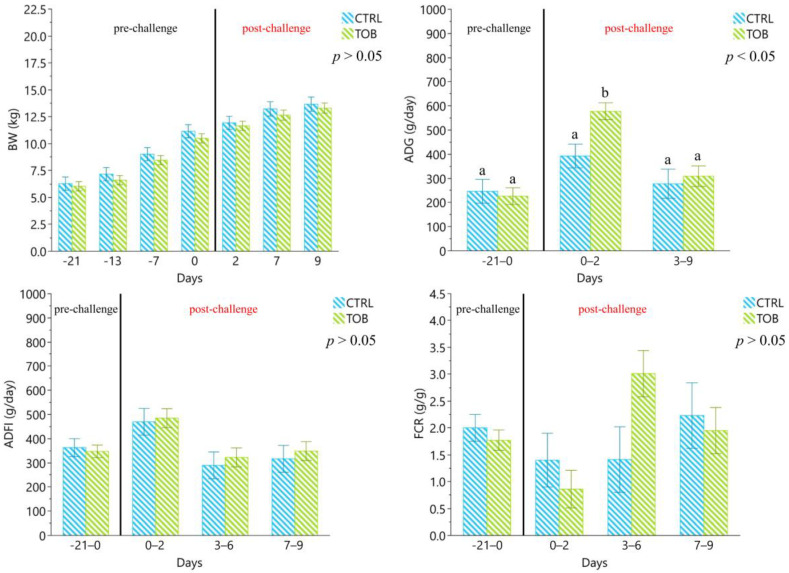
Zootechnical performance from 0 to 29 days of the experimental trial. UC: unchallenged control group, UT: unchallenged treatment group, CC: challenged control group, CT: challenged treatment group. ^a–b^ Different lowercase letters indicate statistically significant differences (*p* < 0.05). The results are presented as least squares means ± standard error.

The body weight (BW), average daily gain (ADG), average daily feed intake (ADFI) and feed conversion ratio (FCR) did not differ during the immunization and post-challenge period for all the experimental groups (Figure 1).

The clinical scores showed a significant increase in the sum of epiphora, oedema, vitality, depression, hair and faecal scores during the post-challenge period (*p* < 0.05; Table 1). The average sum of the epiphora score was significantly higher in the CC group compared to the UC and UT groups (CC: 2.83 ± 0.35; UC 1.33 ± 0.50; UT: 0.50 ± 0.50; *p* < 0.01), while the CT group did not reveal statistical differences during the period from 20 to 23 days. The average sum of epiphora was significantly higher in the CC group compared to the UT and CT groups during the post-challenge period (CC: 8.38 ± 1.06; UT: 1.75 ± 1.51; CT: 4.00 ± 0.06; *p* < 0.01). The average sum of the oedema and hair scores was significantly higher in the CC group compared to the UT and CT groups from days 20 to 23 post-challenge (*p* < 0.01). The average of the oedema scores was increased in the CC group compared to the other experimental groups from 20 to 29 days (CC 9.63 ± 0.52; UC: 3.25 ± 0.74; UT: 0.50 ± 0.74; CT: 1.50 ± 0.52; *p* < 0.01). The vitality scores were found to be higher in the CC group compared to other challenged and unchallenged groups during the period from 20 to 23 days (CC: 1.83 ± 0.16; UC: 0.00 ± 0.23; UT: 0.00 ± 0.23; CT: 0.25 ± 0.16; *p* < 0.01). The average sum of the depression scores was higher in the CC group compared to the unchallenged groups from 20 to 23 days, while the CT group did not show any statistical difference during the same period (CC: 1.33 ± 0.18; UC: 0.33 ± 0.27; UT: 0.33 ± 0.27; CT: 0.75 ± 0.18; *p* < 0.01). From 20 to 29 days, the average sum of the depression scores was elevated in the CC group compared to all the other experimental groups (CC: 5.00 ± 0.67 ^b^; UC: 0.25 ± 0.95; UT: 0.00 ± 0.95; CT: 0.63 ± 0.67; *p* < 0.01). The average sum of the hair scores was significantly lower in the UT and CT groups compared to the CC group, and the UC group did not highlight any statistical difference from 20 to 29 days (UC: 6.75 ± 1.45; UT: 2.50 ± 1.45; CC: 11.50 ± 1.02; CT: 4.50 ± 1.02; *p* < 0.01). The average sum of the faecal scores was increased during the period from 20 to 23 days between the CC and CT groups (CC: 5.33 ± 0.60; CT: 3.00 ± 0.60; *p* = 0.0507).

### 2.2. Microbiological Analyses

**Figure 2:**
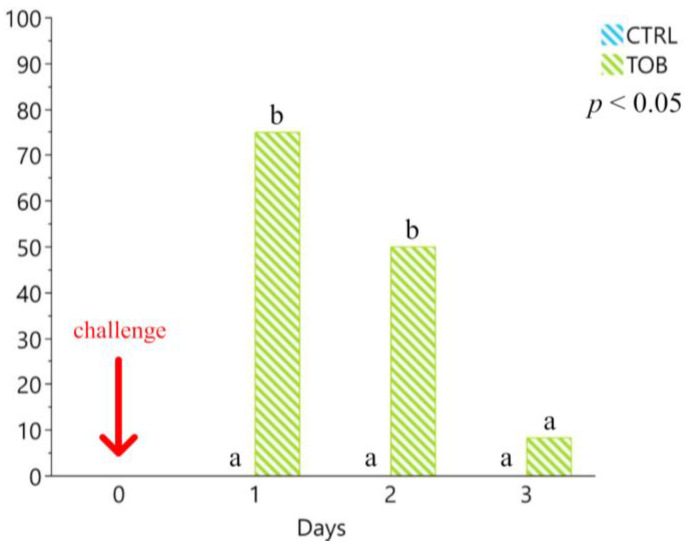
Faecal shedding of the O138 *E. coli* challenge strain after experimental infection for the challenged control (CT) and the challenged treatment (UT) groups during the first five days post-challenge. The experimental infection was represented by a single intragastric inoculum (5 mL) equivalent to 1 × 10^10^ O138 F18 *E. coli* colony-forming units (CFU)/5 mL and was performed at day 20. The arrow indicates the day of infection. The results are presented as percentages of positive animals per total animals per group for each day. ^a–b^ Different lowercase letters indicate statistically significant differences (*p* < 0.01). UC: unchallenged control group, UT: unchallenged treatment group, CC: challenged control group, CT: challenged treatment group. The results are presented as a percentage of positive samples per total animals for each group.

Before the experimental infection procedures, no positivity was detected for the presence of O138 *E. coli* strains in the pigs’ faeces. Faecal shedding of O138 *E. coli* was recorded from 21 to 25 days in the infected groups (CC and CT; Figure 2). In particular, the prevalence of faecal shedding of O138 *E. coli* was significantly higher at 23 days in the CC group compared to the CT group (33.33% and 0.00%, respectively; *p* < 0.01). The CC group showed a significantly longer period of prevalence of challenge strain faecal shedding. In particular, 33% of the piglets showed faecal shedding for 4 consecutive days, while no piglets showed more than 3 days of shedding of O138 *E. coli* in the CT group (*p* < 0.01).

### 2.3. Histological Evaluations 

**Figure 3:**
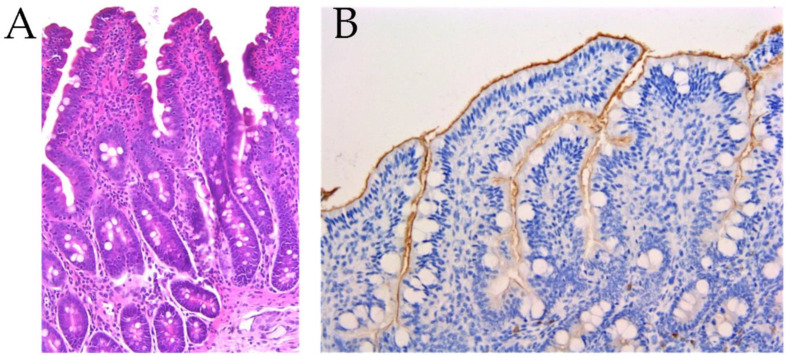
Histological sections of ileum samples at day 23. (**A**): Hematoxylin eosin stain of an ileum section of the challenged control group (CC), 200×. The presence of numerous inflammatory cells (lymphocytes, histiocytes, eosinophils) in the lamina propria of villi. (**B**): Immunohistochemistry for IgA in an ileum section of the unchallenged tobacco vaccinated group (UT), 200×. A positive signal of the presence of IgA is localized on the luminal surface of the enterocytes.

The histological findings in the ileum did not reveal statistical differences among the experimental groups at 23 days (Table 2; Figure 3). Even if infiltrates of lymphocytes were found to be significant at 29 days, only the CC group tended to have increased levels compared to the UC group (*p* < 0.09). The expression of CD3 in the epithelium was significantly different among the experimental groups after 29 days (*p* = 0.0396). However, pairwise comparisons did not highlight any significant difference. Histology and immunohistochemistry showed significant differences within the experimental groups when comparing 23 to 29 days of the experimental period (Table 3; *p* < 0.05). In particular, infiltrated lymphocytes and CD3 in the lamina propria were decreased after 29 days as compared to 23 days in the UC group (*p* = 0.05). Secretory IgA(SIgA) levels in the luminal surface were elevated at 23 days compared to 29 days in the UT group (2.00 ± 0.00 and 0.00 ± 0.00, respectively; *p* = 0.05) Follicular hyperplasia was significantly higer in the CC group at 23 days compared to 29 days (0.50 ± 0.16 and 0.00 ± 0.11, respectively; *p* < 0.05).

### 2.4. Immune Response Parameters

Specific anti-F18 IgAs showed a significant increase in the UT and CT immunized groups compared to the CC group (*p <* 0.05) (Table 4). Relative expression measured by real-time PCR and ELISA quantification did not reveal statistical differences for MHC-I, MHC-II, IFN-γ, IL-1β, TLR2, TLR4, serum IgA, IgA in an intestinal scrape, CXCL9 in an intestinal scrape, TNF-α in an intestinal scrape, IL-8 in an intesinal scrape or IL-1 in an intestinal scrape between the groups and comparing day 3 with day 9 post-challenge (Table 5 and Table 6).

## 3. Discussion

This study aimed to exploit an engineered tobacco seeds-based oral vaccine against VTEC O138 *E. coli* in weaned piglets. The animals enrolled in the study were susceptible to *E. coli* strains harbouring F18 fimbriae as proven by screening for FUT1 polymorphism. 

The pigs in the vaccinated group were fed with 10 g of F18 (7 µg of F18 protein) and 10 g of VT2e-B (containing about 35 µg of VT2e-B), while the animals in the control group received 20 g of wild-type tobacco seeds.

The zootechnical performance did not show statistical differences among the challenged and unchallenged groups during the entire experimental period.

A clinical evaluation revealed significant differences between the groups after the experimental infection. The typical symptoms related to O138 *E. coli* infections are mostly caused by bacterial verotoxins and F18 adhesive fimbriae, which can impair the vascular endothelium of the small intestine, subcutis and brain and eventually cause subcutaneous oedema and neurological symptoms, such as convulsion, rear-leg ataxia, extensor rigidity, lateral recumbency, tremors, paralysis, dyspnea, opisthotonos and sudden death [16]. The infected groups showed a broad spectrum of clinical symptoms, such as epiphora, oedema and loss of vitality due to the *E. coli* challenge.

In particular, the average sum of the epiphora scores was found to be higher in the CC group compared to the UT group 3 days post-challenge. At 9 days post-infection, the average of the sum of the epiphora scores was increased in the CC group compared to the UT and CT groups. The average sum of the oedema scores was lower in the UT and CT groups compared to the CC group during the first three and nine days post-infection. The average of sum of the vitality scores registered a higher value (2 = bad) in the CC group compared to the other experimental groups three days post-challenge. Depression registered decreased scores in the UC and UT groups compared to the CC group three days post-challenge and at nine days, the CC group showed the highest values compared to the other groups.

The average sum of the hair scores revealed increased values in the CC group compared to the UC and UT groups 3 days post-infection. The piglets in the CC group displayed the highest values for hair scores when considering the nine days post-challenge.

The CC group expressed a plethora of clinical symptoms (specific and nonspecific) related to VTEC infection, displaying impaired health conditions during the 9 days post-infection. The CT group revealed values that were comparable to the unchallenged groups, suggesting the preventive effect of the oral vaccination that was able to limit the detrimental effect of the VTEC infection on animal health. In line with the previous study [15], the clinical evaluation allowed us to identify a panel of clinical symptoms related to VTEC infection that are useful in assessing the effectiveness of an edible vaccination.

The faecal score showed increased frequency of diarrhoea in the CC group compared to the CT group during the first 3 days post-challenge. Diarrhoea is the most evident clinical symptom related to VTEC infection and is caused by an alteration of the electrolyte homeostasis, contributing to fluid losses [17]. The results obtained indicate that oral vaccination with tobacco seeds limited diarrhoea occurrence during the first days after infection, underlining the role of vaccination as an intervention to reduce the need for antibiotic treatment [18]. 

The presence of O138 *E. coli* infection was verified by the faecal shedding of the pathogenic challenge strain. The CC and CT groups showed the presence of the pathogen, thus confirming the success of the experimental challenge procedure. However, faecal shedding of the O138 *E. coli* strain was significantly lower in the CT group compared to the CC group 3 and 4 days post-infection. These findings suggest that oral vaccination significantly reduced the number of days of faecal shedding of pathogenic strains. Such reduction of both pathogen excretion and shedding duration should be considered as a successful result of an alternative approach to the use of antibiotics [19].

A histological assessment of the intestinal sections showed significantly higher infiltrates of lymphocytes in the CC group compared to the UC group after 29 days of the trial. The detection of lymphocytic infiltrate indicates the presence of a triggering stimulus able to induce the pathological process. It is well known that enteric pathogens can induce an inflammatory response in the gut [20]. The CC group showed higher follicular hyperplasia compared to the UC group 9 days post-challenge. Follicular hyperplasia is generally the result of accumulation of plasma-cell in lymph nodes and is often characterized by a significant enlargement in one or several segments of the gastrointestinal tract or by a similar alteration of the lymphoid nodules of the Peyer’s patches of the distal part of the small intestine [21]. In general, intestinal bacterial infections cause an increased number of lymphoid follicles [22]. The detected higher follicular hyperplasia in the CC group after 23 days of the trial might be due to virulence factors of the challenger strain that triggered the immune and inflammatory response, which was also confirmed by the highest cases of diarrhoea recorded in the same period. Nevertheless, the CT group showed comparable levels of lymphocytic infiltrate and follicular hyperplasia with the unchallenged groups, suggesting that the tobacco vaccination limited the detrimental effect of the challenge strain by regulating the intestinal inflammatory status. The piglets in the UC group displayed decreased levels of infiltrates of lymphocytes and CD3 in the lamina propria at 29 days compared to 23 days post-challenge. 

The evaluation of specific anti-F18 IgAs showed that the vaccinated groups developed a higher concentration of immunoglobulins A compared to the CC group. In particular, the UT group displayed higher levels of specific anti-F18 IgAs compared to the CC groups. In addition, the UT group showed an increased presence of IgAs in the luminal surface at 23 days compared to 29 days post-challenge. A tendency toward the same trend was found in the UT group for IgAs in the intestinal crypts. Immunoglobulin A (IgA) is the predominant antibody isotype in the mucosal immune system, which widely exists in the gastrointestinal tract, respiratory tract, vaginal tract, tears, saliva and mammary gland [23]. This form of immunoglobulin can be secreted by different mucosal tissues in the body, and they can prevent initial pathogen attachment to the epithelium and subsequent entry [24]. The observed increase in specific anti-F18 IgAs and SIgAs in the luminal surface in the vaccinated groups compared to the CC group suggest that oral vaccination stimulated a protective response against VTEC infection. Mucosal surfaces serve as the primary portal of entry for most pathogens. The mucosal vaccination by edible vaccines provides a safe and efficient mechanism to induce mucosal and systemic immunity against pathogens [25]. Our findings are in agreement with those reported by other authors that indicate that oral vaccination mainly increases the secretory immunoglobulin A concentrations, promoting the local immune response [26,27,28]. The data obtained with this study indicate that oral vaccination with 10 g of F18 engineered tobacco seeds containing 7 µg of recombinant antigen may trigger the specific immune response by increasing the production of specific anti-F18 IgAs.

Compared to a previous study, the oral vaccination with tobacco seeds resulted in effective lowering of the clinical scores, decreasing of the faecal score and O138 *E. coli* shedding duration after the experimental infection [12]. In addition, the engineered tobacco seeds stimulated the immune response by increasing the concentrations of specific anti-F18 IgAs and SIgAs in the luminal surface in the vaccinated groups, indicating that oral vaccination efficiently stimulated the immune system against O138 *E. coli*.

In conclusion, our findings encourage scientific studies aimed to develop innovative prophylaxis tools based on engineered plants. Edible vaccines are interesting and may become fundamental for the sustainable development of a livestock system aimed at improving animal health and reducing the use of antibiotics. 

## 4. Materials and Methods

### 4.1. Production and Characterization of Engineered Tobacco Seeds

In this study, three lines of tobacco seeds *(Nicotiana tabacum L., cv. Xanthi*) were considered: (i) engineered seeds expressing Fed-A, the major subunit of the F18 adhesive fimbriae of verocytotoxic O138 *E. coli* strain (F18s); (ii) engineered seeds expressing the B subunit of the verocytotoxin type 2 (VT2e) toxin of the verocytotoxic O138 *E. coli* strain (VT2eBs); (iii) wild-type tobacco seeds as controls (WTs). The engineered tobacco seeds were previously obtained by stable transformation of tobacco plants via *Agrobacterium tumefaciens* with chimeric constructs containing structural parts of bacterial genes under control of a seed-specific GLOB promoter [29]. The three lines of the experimental seeds were harvested from tobacco plants that were cultivated separately in a greenhouse under a polyethylene monofilament to avoid cross-pollination and to provide protection against parasites (Orto Botanico “G. Emilio Ghirardi”, University of Milan, Toscolano Maderno, Italy).

The tobacco seeds were evaluated in order to verify the morphology and germination characteristics. Additionally, the presence of antigenic bacterial genes in the engineered seeds and the absence of the exogenous genes in the wild-type lines were verified by PCR using specific primer pairs (Table 7).

The PCR assays were performed in 25 μL volume using 5 μL of a DNA template, 0.2 mM of deoxynucleotide triphosphates, 2.5 μL of 10× PCR buffer II, 3 mM MgCl_2_, 1 μM concentrations of each forward and reverse primer and 1.25 U of AmpliTaq Gold DNA Polymerase (Perkin Elmer, Norwalk, CT, USA). The thermal cycling conditions were set up as follows: initial denaturation at 95 °C for 5 min, followed by 35 cycles of denaturation at 95 °C for 1 min, annealing at 56 °C for 1 min and 20 s, elongation at 72 °C for 1 min and 30 s and a final extension at 72 °C. The total proteins were obtained from mature transformed tobacco seeds (from each line) by homogenization with liquid nitrogen in a mortar and extracted with the extraction buffer (50 mM Tris, pH 8.5 mM EDTA, 200 mM NaCl, 0.1% Tween 20). The seed expression of the major subunit Fed-A adhesive fimbriae was evaluated in F18s by indirect ELISA using anti-F18 antibody produced by GenScript (GenScript 860 Centennial Ave. Piscataway, NJ, USA) and purified F18 antigens [30] as a positive control. The seed expression of the B subunit of the VTe2 toxin was evaluated in the VTe2Bs by indirect ELISA using anti-VT2e-B polyclonal antibodies purchased from Plantechno s.r.l (Casalmaggiore, CR, Italy) and VT2eB protein expressed by the pET-system (Novagen) in *E. coli* BL21 as described by Rossi et al. [29]. 

### 4.2. Animals and Housing Conditions 

Thirty-six piglets (Landrace × Large White) were enrolled from a conventional herd free from diseases according to the ex-A-list of the World Organization for Animal Health, and from Aujeszky’s disease, atrophic rhinitis, transmissible gastroenteritis, porcine reproductive and respiratory syndrome and salmonellosis, without history of post-weaning diarrhoea (PWD) and oedema disease (OD) and after a bacteriological analysis of faeces negative for hemolytic *Escherichia coli*. 

Piglets susceptible to verocytotoxic O138 *E. coli* were selected for the polymorphism of a 1,2 fucosyltransferase (FUT1) gene by PCR performed on genomic DNA extracted from duodenum tissue using the Wizard^®^ Genomic DNA Purification Kit (Promega Italia S.r.l., Milan, Italy), according to previous study [31]. 

The piglets, homogeneous for weight, age and sex (50% male, 50% female), weaned at 21 ± 2 days (to ensure the animals’ sensitivity upon experimental infection) were transported to the Experimental Animal Research and Application Centre (pigs sector) of the University of Milan in Lodi.

The animals, identified by auricular tag, were housed in individual pens (100 × 50 cm) under controlled conditions (27–29 °C; 60% of relative humidity) with ad libitum access to water, feed and environmentally safe chewable enrichment materials. 

The animals were fed basal diets in line with the nutritional requirements of weaned piglets [32] without antibiotics, with 50 ppm of zinc provided by Ferraroni S.p.A (Bonemerse CR, Italy). From the day of the challenge until the second day after the challenge, the same antimicrobial-free diet, containing 27% of crude protein on dry matter, was administered to all the experimental groups, according to Rossi et al. [15].

The experimental diets and tobacco seeds were analysed for the principal nutrient components following the official method of analysis of the Association of Analytical Chemists [33]. In particular, the following parameters were determined: dry matter (DM; method 930.15), crude protein (CP; method 2001.11); ether extract (EE; method 2003.05); crude fiber (CF; method Ba 6a-05) by the filtering bags technique, following the official method of the American Oil Chemists Society [34]; and ash (method 942.05). The composition of the diets is reported in Table 8.

The piglets were randomly divided into two experimental groups: the control group (C; *n* = 18) and the treatment group (T; *n* = 18).

### 4.3. Experimental Design and Treatments

The study was divided into three phases: (i) oral immunisation (days 0–20): administration of the engendered seeds (vaccine) by oral route on days 0, 1, 2, 5 and 14; (ii) challenge (day 20): experimental infection of 50% of the animals; (iii) observation (days 21–29): clinical and zootechnical evaluations and collection of samples. The study was performed single blinded.

The tobacco seeds were ground with a mill to obtain a uniform powder using a 0.75 mm grid under controlled temperature (max 50 °C). During the immunization phase (on days 0, 1, 2, 5 and 14), the tobacco seeds were administered with a commercial milk replacer powder (LACTOSOL) by mixing in a 1:2 ratio (*w*/*w*) in order to guarantee complete consumption. The specific pig milk replacer powder was composed of dairy products, vegetable oils, cereals, sugars and flavouring, containing 20.5% crude proteins.

The T group received *Nicotiana tabacum* seeds (F18s + VT2e-Bs) as a vaccine on days 0, 1, 2, 5 and 14 by feeding 10 g of F18s and 10 g of VT2e-B mixed with 40 g of milk replacer powder. On the same experimental days, the C group received 20 g of wild-type milled tobacco seeds mixed with 40 g of milk powder. 

### 4.4. Challenge 

Six days after a booster immunization, on day 20, 24 piglets (12 animals per group) were orally challenged with the O138 *Escherichia coli* strain, which was provided by the Experimental Zooprophylactic Institute of Lombardy and Emilia Romagna (IZSLER). 

One hour before the challenge, the piglets were intramuscularly injected with azaperone (2 mL/pig, Stresnil^TM^, Janssen Cilag SpA, Milan, Italy) and 30 mL of a 10% NaHCO_3_ solution was orally delivered to increase the challenge strain viability. After 10–15 min, a single dose of 5 mL of 10^10^ CFU/mL of the O138 *E. coli* challenge strain was given through an intragastric catheter (16 G), according to Rossi et al. [15]. The animals fasted 3 h before and 3 h after challenge.

**Figure 4:**
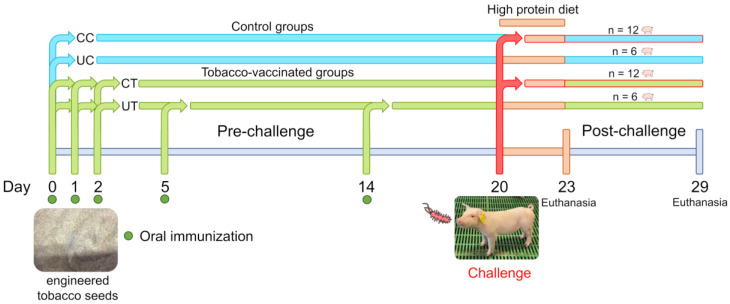
Experimental design from 0 to 29 days divided by immunization phase (pre-challenge) and post-challenge. C: control group fed wild-type tobacco seeds, T: treatment group fed engineered tobacco seeds, CC: challenged control group, UC: unchallenged control group, CT: challenged treatment group, UT: unchallenged treatment group.

For each group, six animals were not subjected to the challenge and were separated from the infected pigs by applying biosafety barriers (two empty pens for the separation), changing gloves, boot covers and plastic coats during the passage of operators from the unchallenged to the infected groups. The animals were then divided into four experimental groups for the post-challenge phase as unchallenged control (UC), challenged control (CC), unchallenged treatment (UT) and challenged treatment (CT; Figure 4).

### 4.5. Zootechnical and Clinical Evaluation and Sampling

All the piglets were individually weighed weekly during the entire experimental period and twice a week during the post-challenge period (25 and 29 days); the feed intake (FI) was measured on a daily basis by weighing the feed refuse per pen (experimental unit for FI evaluation). In the same period, faecal consistency was evaluated daily through a four-point scale: 0 = normal faeces; 1 = soft consistency 2 = mild diarrhoea; 3 = severe diarrhoea [35].

Blood samples were collected from the jugular vein of each animal to determine the haematocrit value at 0, 7, 14, 21, 25 and 29 days. During the pre-challenge period, faecal samples were taken from the rectum weekly on 0, 7, 14 and 21 days to determine the total and specific IgA levels, as described below. 

During the post-challenge period, faecal samples were collected in order to evaluate the shedding of the challenge strains. A rectal temperature was recorded daily. Clinical signs of VTEC infection were registered daily according to the adopted translational model [15]. In particular, palpebral oedema, epiphora, vitality, respiratory and neurological problems were monitored daily and scored using specific point scales. Epiphora is an overflow of tears due to the inflammation and the reduced motility of the eyelid or of the lacrimal pump and was scored as follows: 0 = normal; 1 = mild (moderate presence of eye discharge material in the corner of the eye); 2 = severe (abundant presence of brown discharge material in the corner of the eye). Palpebral oedema is defined as a pathological condition showing an accumulation of fluids in the inner part of the eyelids with different degrees of severity according to the adopted scoring: 0 = normal; 1 = mild (puffy eyelids giving a sleepy appearance); 2 = severe (prominent and closed eyelids). Vitality and depression were defined as disturbed behaviour or slow reactions, an unsteady and slow gait whilst walking and an inattentive response when encouraged to move. Vitality score: 0 = good; 1 = loose (failure to react to stimulus); 2 = bad (slow response to stimuli); depression score: 0 = high (lethargy); 1 = mild (slow reactions); 2 = normal status; perineal area: 0 = clean; 1 = smear; 3 = smear with flogosis (red area caused by inflammation); respiratory score: 0 = normal; 1 = slightly quick; 2 = quick; neurological score: 0 = normal; 1 = mild symptoms (incoordination); 2 = severe symptoms (lateral position, paddling limbs, central nervous symptoms); hair score: 0 = regular (smooth, clean, flat and uniform); 1 = slightly irregular (fuzzy hair coat and/or scaly skin); 2 = irregular (bald patches, or a rough, dull, uneven coat and reddened skin). 

At day 3 post-challenge (23 days), 12 piglets (CC = 4, UC = 2, TC = 4, UT = 2) were euthanized for post-mortem examination; the other animals were sacrificed at 29 days (9 days post-challenge). Jejunum, mesenteric lymph nodes and intestinal scrapes were collected and stored in RNA Later (Quiagen, Hilden, Germany) for gene expression analysis. A part of the ileum samples was frozen for immunochemistry, and another one was fixed in 10% neutral buffered formalin for immunohistochemistry and histological evaluations. The samples of the ileum were collected and sealed with sterile cotton ligatures, 3 cm apart from the proximal and distal ends and used for microbiological analyses.

### 4.6. Microbiological Analysis

The faecal samples were analysed for animal selection and to confirm the success of experimental challenge procedures. A total of 1 g of faeces was homogenized in 1 mL of sterile saline buffer and plated onto 5% sheep blood agar plates (blood agar base no. 2, Oxoid, Altrincham, UK) and incubated overnight at 37 °C to assess the presence of haemolytic colonies. Up to 5 haemolytic colonies were selected from each plate and seeded on MacConkey agar (Oxoid, Altrincham, UK), triple sugar iron agar (Oxoid, Altrincham, UK), Simmons citrate agar (Oxoid, Altrincham, UK), and buffered peptone water broth (Oxoid, Altrincham, UK). The bacterial colonies positive for glucose oxidation–fermentation, fermentation of lactose, indole production and sodium citrate-negative were tested with the API system^®^ (API 20 NE—BioMerieux, Marcy-l’Étoile, France) in order to obtain a more precise identification. *Lactobacillus* spp. and *Enterobacteriaceae* were identified on adequate selective media and incubated following adequate conditions. The bacterial concentrations were determined with a semi-quantitative approach based on serial dilutions with medium (detection limit 1 CFU/g).

The *Escherichia coli* strains isolated from the faecal samples that were cultured on blood agar and MacConkey were further isolated on trypticase soy agar (Oxoid, Altrincham, UK) at 37 °C for 24 h and biochemically identified with API-20E method (BioMerieux, Marcy-l’Étoile, France). Besides the evaluation of haemolytic activity, the *E. coli* were serotyped by using monospecific antisera for the O138 antigen and genetically characterized by PCR for Vt, Vt1, Vt2 and Vt2e. The Vt2e positive strains were assessed for the presence of the F18 gene.

### 4.7. Histological Evaluations 

The organs fixed in formalin were paraffin wax-embedded, sliced with a microtome into 5 μm thick histological sections, stained with haematoxylin and eosin for 10 min and microscopically examined at 200× and 400× optical magnifications. The intestinal samples were semi-quantitatively scored (0 = absent, 1 = slight, 2 = moderate, 3 = strong) for the presence of inflammation in the villi and lamina propria (infiltrates of lymphocytes, plasma cells, histiocytes and eosinophils), epithelial regeneration (enterocytes with high nucleus/cytoplasm ratio on the intestinal layer), fusion of the villi, oedema in the deep lamina propria and T atrophy (T-dependent hypocellularity areas), stroma (fibroconnective and histiocytes) and follicular hyperplasia. 

For the immunohistochemistry assays, the tissues were dewaxed and unmasked by heat-induced epitope retrieval and Buffer H (Bio-Optica, Milan, Italy); the endogenous peroxidase activity was blocked by incubation with 3% H_2_O_2_. After rinsing and treating with PBS (with normal serum to reduce nonspecific background staining), the sections were incubated with primary antibodies specific for Iba1 (Wako, Neuss, Germany), CD3 (Dako, Næstved, Denmark), CD20 (Thermo Scientific, Rome, Italy), IgG (Vector Laboratories, Burlingame, CA, USA) and IgA (Abcam, Cambridge, UK) at the suggested dilutions. The samples were subsequently incubated with biotinylated secondary antibody (rabbit anti-goat, Vector Laboratories, Burlingame, CA, USA) and labelled by the avidin-biotin-peroxidase (ABC) procedure (VECTASTAIN^®^ Elite ABC-Peroxidase Kit Standard, Vector Laboratories, Burlingame, CA, USA). The immunoreaction was visualized with a 3,3′-diaminobenzidine substrate (Peroxidase DAB Substrate Kit, Vector Laboratories, Burlingame, CA, USA) after counterstaining with Mayer’s haematoxylin (Merck, Kenilworth, NJ, USA) and blindly evaluated with a light microscope by a veterinary pathologist. The known positive control sections were included in each assay. The CD3, CD20 and Iba1 immunoreactions were semi-quantitatively scored in the lamina propria (0 = absent; 1 = rare cells; 2 = some cells; 3 = numerous cells; 4 = very numerous cells), while the IgG and IgA immunoreactions were semi-quantitatively scored on the epithelial luminal surface and lamina propria (0 = absent, 1 = slight, 2 = moderate; 3 = strong).

### 4.8. Evaluation of Specific Intestinal Immunoglobulin-A Titer 

For the evaluation of specific IgA (anti-F18 and anti-VT2eB), a double antibody sandwich ELISA system was developed. Fresh faeces (1 g) were vortexed in 10 mL of extraction buffer (0.01M PBS, 0.5% Tween, 0.05% sodium azide) and centrifugated at 1500× *g* for 20 min at 4 °C. A total of 2 mL of the supernatant were transferred to a sterile tube containing 20 µL of protease inhibitor (Roche Diagnostics, Mannheim, Germany) and mixed briefly. Finally, the samples were centrifuged at 10,000× *g* for 10 min and the supernatant was collected in sterile tubes. 

The anti-pig IgA antibodies (Bethyl 1:500 in coating buffer, 50 mM sodium carbonate pH 9.6) were coated in a 96-well plate overnight at 4 °C. The plates were blocked with PBST and 3% BSA for 3 h at room temperature. The total immunoglobulins extracted from the faeces (included those linked to F18 and VT2eB) were incubated overnight at 4 °C in microtiter wells. After three washes with PBST, 100 µL of anti-F18 or anti-VT2eB rabbit IgGs were added to each well and incubated for 1 h at room temperature. After three washes with PBST, 100 µL of anti-rabbit IgG horseradish peroxidase (HRP) conjugate antibodies were added to each well for 1 h at room temperature. TMB substrate was used for the colorimetric reaction. The reaction was stopped by the addition of 50 µL of stop solution (H_2_SO_4_ 0.16 M). The absorbances were measured spectrophotometrically by a microplate reader at 450 nm (Model 680 Microplate Reader, Bio-Rad Laboratories Inc., Hercules, CA, USA). The data were expressed as optical densities (O.D.).

### 4.9. Immunoenzymatic Evaluation

The intestinal scrapes were homogenized using a rotor-stator system (Ultra Turrax T25, Staufen im Breisgau, Germany) in a lysis buffer (50 mM Tris–HCl, pH 7.4, 1% Nonidet P-40, 0.25% sodium deoxycholate, 150 mM NaCl, 1 mM EDTA, Sigma-Aldrich, Saint Louis, MO, USA) with a protease inhibitor cocktail (1 mM PMSF, 5 μg/mL Complete Protease Inhibitor Cocktail, Roche Diagnostics, Mannheim, Germany) and centrifuged at 470× *g* for 15 min at 4 °C. The supernatants were collected, and the protein content was quantified by direct absorbance measurement at 280 nm in a quartz cuvette.

The titres of the serum and intestinal mucosa total IgA were determined using specific immunoenzymatic kits (Swine IgA ELISA Quantitation Set, Bethyl Laboratories Inc., Montgomery, TX, USA) according to the manufacturer’s instructions. The intestinal mucosa TNF-α, IL-8 and CXCL9 (MIG) levels were measured using three specific ELISA kits (Kingfisher Biotech Inc., St. Paul, MN, USA), while the intestinal mucosa IL-1β was determined using a porcine interleukin 1β ELISA kit (Cusabio Life Science, Houston, TX, USA). Negative and blank (buffer) samples were included on each plate along with duplicates of each sample.

### 4.10. RNA Extraction, Reverse Transcription and Real-Time PCR Assays

The total RNA was isolated from the homogenized samples by a rotor–stator system (Ultra Turrax T25, Staufen im Breisgau, Germany) in 2 mL of TRI^®^Reagent (Sigma-Aldrich, Saint Louis, MO, USA). The purity and concentration of the total RNA were spectrophotometrically evaluated (BioPhotometer Eppendorf, Hamburg, Germany) and 1 µg of total RNA from each sample was reverse transcribed to cDNA using the High-Capacity cDNA Archive kit (10 min at 25 °C, 60 min at 37 °C and 5 min at 95 °C) (Applied Biosystems, Waltham, MA, USA). The resulting cDNA was used for real-time PCR (7000 Sequence Detection System, Applied Biosystem, Waltham, MA, USA), according to a published protocol [36], to quantify the expression of the best-characterized swine innate immunity receptors TLRs (2 and 4) and pro-inflammatory cytokines IFN-γ and IL-1β in the jejunum, and the expression of the antigen-presenting molecules MHC (type I and II) in the mesenteric lymph node cells. All the primers were custom synthesized (Invitrogen, Waltham, MA, USA) and designed using the NCBI nucleotide sequences database (Table 9). The porcine housekeeping gene beta-actin was used as an endogenous control.

### 4.11. Statistical Analysis

The zootechnical performance data were analysed using a mixed model, including the fixed effect of time, treatment, their interaction (treatment × time) and the random effect of animals using JMP^®^ Pro 15 (SAS Inst. Inc., Cary, NC, USA). Pairwise post-hoc comparisons were performed using the Tukey’s honestly significant difference test (Tukey’s HSD) or the Tukey–Kramer test, according to the sample size of the group. The faecal *E. coli* shedding prevalence was analysed using the chi-squared test. Other data were analysed using the Kruskal–Wallis test for unpaired samples and separating medians by the Wilcoxon test for each pairwise comparison. The differences were tested firstly between the control and the treated animals. Furthermore, the differences between animals sacrificed at 3 days and 9 days were verified within the control and treated groups, respectively. Finally, the differences between the control and treated animals sacrificed at 3 days and 9 days were also tested. All the measurements were assessed using the individual pig as an experimental unit. The statistical differences were considered when *p* ≤ 0.05. The results were presented as least squares means ± standard error.

## Figures and Tables

**Table 1:** Averages of the sums of the clinical scores of from 20 to 23 days (Σ3) and from 20 to 29 days (Σ9) during the post-challenge period.

	UC	UT	CC	CT
Epiphora_Σ3 *	1.33 ± 0.50 ^ab^	0.50 ± 0.50 ^a^	2.83 ± 0.35 ^b^	1.83 ± 0.35 ^ab^
Epiphora_Σ9 *	5.75 ± 1.51 ^ab^	1.75 ± 1.51 ^a^	8.38 ± 1.06 ^b^	4.00 ± 0.06 ^a^
Oedema_Σ3 *	1.17 ± 0.35 ^ab^	0.33 ± 0.35 ^a^	2.17 ± 0.25 ^b^	1.17 ± 0.25 ^a^
Oedema_Σ9 *	3.25 ± 0.74 ^a^	0.50 ± 0.74 ^a^	9.63 ± 0.52 ^b^	1.50 ± 0.52 ^a^
Vitality_Σ3 *	0.00 ± 0.23 ^a^	0.00 ± 0.23 ^a^	1.83 ± 0.16 ^b^	0.25 ± 0.16 ^a^
Vitality_Σ9	0.00 ± 1.52	0.50 ± 1.52	4.13 ± 1.07	1.00 ± 1.07
Depression_Σ3 *	0.33 ± 0.27 ^a^	0.33 ± 0.27 ^a^	1.33 ± 0.18 ^b^	0.75 ± 0.18 ^ab^
Depression_Σ9 *	0.25 ± 0.95 ^a^	0.00 ± 0.95 ^a^	5.00 ± 0.67 ^b^	0.63 ± 0.67 ^a^
Hair_Σ3 *	1.50 ± 0.32 ^ab^	0.50 ± 0.32 ^a^	1.75 ± 0.23 ^b^	0.50 ± 0.23 ^a^
Hair_Σ9 *	6.75 ± 1.45 ^ab^	2.50 ± 1.45 ^a^	11.50 ± 1.02 ^b^	4.50 ± 1.02 ^a^
Perineal area_Σ3	0.67 ± 0.29	0.50 ± 0.29	0.92 ± 0.21	0.42 ± 0.21
Perineal area_Σ9	1.75 ± 1.14	0.75 ± 1.14	3.75 ± 0.81	1.13 ± 0.81
Faecal score_Σ3 *	4.33 ± 0.85 ^ab^	2.83 ± 0.85 ^ab^	5.33 ± 0.60 ^b^	3.00 ± 0.60 ^a^
Faecal score_Σ9	9.00 ± 2.49	3.00 ± 2.49	11.62 ± 1.76	7.13 ± 1.76

Σ3: sum of the individual daily scores from days 20 to 23 (*n* = 36); Σ9: sum of the individual daily scores from days 20 to 29 (*n* = 24). The comparisons were performed using the Kruskal–Wallis test and the means were separated with the Wilcoxon rank sum test for comparing independent groups. The results are presented as least squares means of the clinical scores sum for each animal per period ± standard error. * The asterisk indicates parameters in which a statistically significant difference was recorded. ^a–b^ Different lowercase letters indicate statistically significant differences (*p* < 0.05). UC: unchallenged control group, UT: unchallenged treatment group, CC: challenged control group, CT: challenged treatment group.

**Table 2:** Mean values of the histological parameters of the ileum sections among the experimental groups recorded at 23 and 29 days.

Day	Analysis	Parameter	UC	UT	CC	CT
23 days	Histology	Infiltrates of lymphocytes	2.00 ± 0.41	n.d.	2.00 ± 0.29	2.00 ± 0.33
		Epithelial regeneration	0.50 ± 0.35	n.d.	0.25 ± 0.25	0.00 ± 0.35
		Fusion of villi	0.50 ± 0.50	n.d.	1.00 ± 0.41	1.00 ± 0.41
		Oedema	0.50 ± 0.57	n.d.	0.25 ± 0.40	0.67 ± 0.67
		T-Atrophy	0.50 ± 0.52	0.50 ± 0.52	0.75 ± 0.37	1.00 ± 0.42
		Stroma	0.50 ± 0.44	0.50 ± 0.44	0.50 ± 0.31	0.67 ± 0.36
		Follicular hyperplasia	0.00 ± 0.39	0.50 ± 0.39	0.50 ± 0.28	0.33 ± 0.32
	Immunohistochemistry	CD3 in epithelium	4.00 ± 0.44	2.00 ± 0.44	2.75 ± 0.31	3.00 ± 0.36
		CD3 in lamina propria	4.00 ± 0.39	3.50 ± 0.39	3.50 ± 0.28	2.67 ± 0.32
		CD20 in epithelium	0.00 ± 0.00	0.00 ± 0.00	0.00 ± 0.00	0.00 ± 0.00
		CD20 in lamina propria	1.00 ± 0.23	1.00 ± 0.23	1.25 ± 0.16	1.00 ± 0.19
		lba1 in villus	4.00 ± 0.32	4.00 ± 0.32	3.75 ± 0.23	3.33 ± 0.26
		lb1 in crypts	2.00 ± 0.37	2.50 ± 0.37	2.25 ± 0.26	2.33 ± 0.30
		IgG in luminal surface	1.00 ± 0.65	1.50 ± 0.65	0.75 ± 0.46	0.33 ± 0.53
		IgG in villus axis	1.00 ± 0.23	1.00 ± 0.23	1.25 ± 0.16	1.00 ± 0.19
		IgG in crypts	2.50 ± 0.42	2.50 ± 0.42	1.75 ± 0.29	2.33 ± 0.34
		SIgA luminal surface	0.50 ± 0.55	2.00 ± 0.55	0.50 ± 0.39	0.67 ± 0.45
		IgA in villus axis	0.50 ± 0.27	0.50 ± 0.27	0.00 ± 0.19	0.00 ± 0.22
		IgA in crypts	1.00 ± 0.48	3.50 ± 0.48	1.75 ± 0.34	2.00 ± 0.39
29 days	Histology	Infiltrates of lymphocytes *	1.00 ± 0.27 ^a^	1.50 ± 0.27 ^ab^	2.13 ± 0.19 ^b^	1.60 ± 0.24 ^ab^
		Epithelial regeneration	0.25 ± 0.38	0.25 ± 0.38	1.00 ± 0.27	0.17 ± 0.31
		Fusion of villi	0.25 ± 0.34	1.00 ± 0.34	1.00 ± 0.24	0.33 ± 0.27
		Oedema	0.25 ± 0.20	0.25 ± 0.20	0.25 ± 0.14	0.00 ± 0.16
		T-Atrophy	0.75 ± 0.31	0.75 ± 0.31	0.25 ± 0.22	0.13 ± 0.22
		Stroma	1.25 ± 0.38	0.75 ± 0.38	0.50 ± 0.27	0.38 ± 0.27
		Follicular hyperplasia	0.00 ± 0.17	0.25 ± 0.17	0.00 ± 0.12	0.25 ± 0.12
		CD3 in epithelium	2.50 ± 0.31	3.50 ± 0.31	3.50 ± 0.22	2.75 ± 0.22
		CD3 in lamina propria	3.00 ± 0.26	2.50 ± 0.26	3.13 ± 0.18	3.25 ± 0.18
	Immunohistochemistry	CD20 in epithelium	0.00 ± 0.00	0.00 ± 0.00	0.00 ± 0.00	0.00 ± 0.00
		CD20 in lamina propria	1.00 ± 0.00	1.00 ± 0.00	1.00 ± 0.00	1.00 ± 0.00
		lba1 in villus	3.75 ± 0.23	3.75 ± 0.23	3.88 ± 0.16	3.63 ± 0.16
		lb1 in crypts	2.25 ± 0.21	2.00 ± 0.21	2.38 ± 0.15	2.13 ± 0.15
		IgG in luminal surface	0.75 ± 0.36	0.00 ± 0.36	0.25 ± 0.25	0.63 ± 0.25
		IgG in villus axis	1.00 ± 0.10	1.00 ± 0.10	1.13 ± 0.07	1.00 ± 0.07
		IgG in crypts	2.25 ± 0.21	2.00 ± 0.21	2.50 ± 0.15	1.88 ± 0.15
		SIgA luminal surface	0.50 ± 0.38	0.00 ± 0.38	0.38 ± 0.27	1.13 ± 0.27
		IgA in villus axis	0.00 ± 0.00	0.00 ± 0.00	0.00 ± 0.00	0.00 ± 0.00
		IgA in crypts	1.75 ± 0.17	2.00 ± 0.17	2.25 ± 0.12	2.00 ± 0.12

Infiltrates of lymphocytes, epithelial regeneration, fusion of villi, oedema, T-atrophy, stroma and follicular hyperplasia were semi-quantitatively scored (0 = absent, 1 = slight, 2 = moderate, 3 = severe). CD3, CD20 and Iba1 immunoreactions were semi-quantitatively scored in the lamina propria (0 = absent, 1 = rare cells, 2 = some cells, 3 = numerous cells, 4 = very abundant cells). For IgG and IgA, the immunoreaction was semi-quantitatively scored in the epithelial luminal surface and lamina propria (0 = absent, 1 = slight, 2 = moderate, 3 = strong). The comparisons were performed using the Kruskal–Wallis test and the means were separated with Wilcoxon rank sum test for comparing independent groups. The results are presented as least squares means ± standard error. * The asterisk indicates parameters in which a statistically significant difference was recorded. ^a–b^ Different lowercase letters indicate statistically significant differences (*p* < 0.05). UC: unchallenged control group, UT: unchallenged treatment group, CC: challenged control group, CT: challenged treatment group.

**Table 3:** Mean values of the histological parameters of the ileum sections recorded at 23 and 29 days divided by experimental groups. *p*-Value most significant in bold.

Group	Analysis	Parameter	Day 23	Day 29	*p*-Value
Unchallenged Control (UC)		Infiltrates of lymphocytes *	2.00 ± 0.00	1.00 ± 0.00	0.0504
		Epithelial regeneration	0.50 ± 0.40	0.25 ± 0.28	0.7799
		Fusion of villi	0.50 ± 0.40	0.25 ± 0.28	0.7799
	Histology	Oedema	0.50 ± 0.40	0.25 ± 0.28	0.7799
		T-Atrophy	0.50 ± 0.64	0.75 ± 0.45	1.0000
		Stroma	0.50 ± 0.64	1.25 ± 0.45	0.4677
		Follicular hyperplasia	0.00 ± 0.00	0.00 ± 0.00	-
		CD3 in epithelium	4.00 ± 0.35	2.50 ± 0.25	0.0902
		CD3 in lamina propria *	4.00 ± 0.00	3.00 ± 0.00	0.0504
		CD20 in epithelium	0.00 ± 0.00	0.00 ± 0.00	-
		CD20 in lamina propria	1.00 ± 0.00	1.00 ± 0.00	-
		lba1 in villus	4.00 ± 0.31	3.75 ± 0.22	0.7237
	Immunohistochemistry	lb1 in crypts	2.00 ± 0.31	2.25 ± 0.22	0.7237
		IgG in luminal surface	1.00 ± 0.77	0.75 ± 0.54	1.0000
		IgG in villus axis	1.00 ± 0.00	1.00 ± 0.00	-
		IgG in crypts	2.50 ± 0.42	2.25 ± 0.21	0.7799
		SIgA luminal surface	0.50 ± 0.66	0.50 ± 0.47	1.0000
		IgA in villus axis	0.50 ± 0.25	0.00 ± 0.18	0.2888
		IgA in crypts	1.00 ± 0.59	1.75 ± 0.41	0.5839
Unchalleged Treatment (UT)		Infiltrates of lymphocytes	n.d.	1.50 ± 0.29	-
		Epithelial regeneration	n.d.	0.25 ± 0.25	-
		Fusion of villi	n.d.	1.00 ± 0.00	-
	Histology	Oedema	n.d.	0.25 ± 0.25	-
		T-Atrophy	0.50 ± 0.64	0.75 ± 0.45	1.0000
		Stroma	0.50 ± 0.64	0.75 ± 0.45	1.0000
		Follicular hyperplasia	0.50 ± 0.39	0.25 ± 0.28	0.7799
		CD3 in epithelium	2.00 ± 0.35	3.50 ± 0.25	0.0902
		CD3 in lamina propria	3.50 ± 0.43	2.50 ± 0.31	0.2113
		CD20 in epithelium	0.00 ± 0.00	0.00 ± 0.00	-
		CD20 in lamina propria	1.00 ± 0.00	1.00 ± 0.00	-
		lba1 in villus	4.00 ± 0.31	3.75 ± 0.22	0.7237
	Immunohistochemistry	lb1 in crypts	2.50 ± 0.25	2.00 ± 0.18	0.2888
		IgG in luminal surface	1.50 ± 0.25	0.00 ± 0.18	0.0552
		IgG in villus axis	1.00 ± 0.00	1.00 ± 0.00	-
		IgG in crypts	2.50 ± 0.25	2.00 ± 0.18	0.2888
		SIgA luminal surface *	2.00 ± 0.00	0.00 ± 0.00	0.0504
		IgA in villus axis	0.50 ± 0.25	0.00 ± 0.18	0.2888
		IgA in crypts	3.50 ± 0.25	2.00 ± 0.18	0.0552
Challenged Control (CC)		Infiltrates of lymphocytes	2.00 ± 0.35	2.13 ± 0.25	0.8481
		Epithelial regeneration	0.25 ± 0.47	1.00 ± 0.33	0.2868
		Fusion of villi	1.00 ± 0.54	1.00 ± 0.33	1.0000
	Histology	Oedema	0.25 ± 0.24	0.25 ± 0.18	1.0000
		T-Atrophy	0.75 ± 0.24	0.25 ± 0.18	0.1371
		Stroma	0.50 ± 0.35	0.50 ± 0.25	0.9229
		Follicular hyperplasia	0.50 ± 0.16 ^a^	0.00 ± 0.11 ^b^	**0.0492**
		CD3 in epithelium	2.75 ± 0.26	3.50 ± 0.19	0.0658
		CD3 in lamina propria	3.50 ± 0.31	3.13 ± 0.22	0.3835
		CD20 in epithelium	0.00 ± 0.00	0.00 ± 0.00	-
		CD20 in lamina propria	1.25 ± 0.14	1.00 ± 0.10	0.2159
		lba1 in villus	3.75 ± 0.20	3.88 ± 0.14	0.6941
	Immunohistochemistry	lb1 in crypts	2.25 ± 0.26	2.38 ± 0.18	0.7559
		IgG in luminal surface	0.75 ± 0.33	0.25 ± 0.23	0.3583
		IgG in villus axis	1.25 ± 0.20	1.13 ± 0.14	0.6941
		IgG in crypts	1.75 ± 0.26	2.50 ± 0.19	0.0658
		SIgA luminal surface	0.50 ± 0.27	0.38 ± 0.19	0.7662
		IgA in villus axis	0.00 ± 0.19	0.00 ± 0.00	-
		IgA in crypts	1.75 ± 0.24	2.25 ± 0.17	0.1461
Challenged Treatment (CT)		Infiltrates of lymphocytes	2.00 ± 0.26	1.60 ± 0.20	0.3241
		Epithelial regeneration	0.00 ± 0.26	0.17 ± 0.15	0.7728
		Fusion of villi	1.00 ± 0.25	0.33 ± 0.18	0.1011
	Histology	Oedema	0.67 ± 0.36	0.00 ± 0.25	0.2386
		T-Atrophy	1.00 ± 0.33	0.13 ± 0.20	0.0903
		Stroma	0.67 ± 0.31	0.38 ± 0.19	0.4795
		Follicular hyperplasia	0.33 ± 0.28	0.25 ± 0.17	0.8952
		CD3 in epithelium	3.00 ± 0.45	2.75 ± 0.28	0.7411
		CD3 in lamina propria	2.67 ± 0.28	3.25 ± 0.17	0.1518
		CD20 in epithelium	0.00 ± 0.00	0.00 ± 0.00	-
		CD20 in lamina propria	1.00 ± 0.00	1.00 ± 0.00	-
		lba1 in villus	3.33 ± 0.31	3.63 ± 0.19	0.4795
	Immunohistochemistry	lb1 in crypts	2.33 ± 0.24	2.13 ± 0.15	0.5428
		IgG in luminal surface	0.33 ± 0.49	0.63 ± 0.30	0.8120
		IgG in villus axis	1.00 ± 0.00	1.00 ± 0.00	-
		IgG in crypts	2.33 ± 0.24	1.88 ± 0.15	0.1731
		SIgA luminal surface	0.67 ± 0.59	1.13 ± 0.36	0.5197
		IgA in villus axis	0.00 ± 0.00	0.00 ± 0.00	-
		IgA in crypts	2.00 ± 0.00	2.00 ± 0.00	-

Infiltrates of lymphocytes, epithelial regeneration, fusion of villi, oedema, T-atrophy, stroma and follicular hyperplasia were semi-quantitatively scored (0 = absent, 1 = slight, 2 = moderate, 3 = severe). CD3, CD20 and Iba1 immunoreactions were semi-quantitatively scored in the lamina propria (0 = absent, 1 = rare cells, 2 = some cells, 3 = numerous cells, 4 = very abundant cells). For IgG and IgA, the immunoreaction was semi-quantitatively scored in the epithelial luminal surface and lamina propria (0 = absent, 1 = slight, 2 = moderate, 3 = strong). The comparisons were performed using the Wilcoxon rank sum test for independent groups. The results are presented as least squared means ± standard error. * The asterisk indicates parameters in which a statistically significant difference was recorded. ^a–b^ Different lowercase letters indicate statistically significant differences (*p* < 0.05). UC: unchallenged control group, UT: unchallenged treatment group, CC: challenged control group, CT: challenged treatment group.

**Table 4:** Titer of specific anti-F18 and anti-VT2 IgAs in faecal samples.

	UC	UT	CC	CT
anti-F18 IgA *	0.87 ± 0.17 ^ab^	1.36 ± 0.15 ^b^	0.65 ± 0.11 ^a^	1.15 ± 0.15 ^b^
anti-VT2IgA	0.89 ± 0.19	1.43 ± 0.11	0.89 ± 0.19	1.37 ± 0.13

The results are presented as least squares means of optical densities ± standard error. The comparisons were performed using the Kruskal–Wallis test and the means were separated with the Wilcoxon rank sum test for comparing independent groups. * The asterisk indicates parameters in which a statistically significant difference was recorded. ^a–b^ Different lowercase letters indicate statistically significant differences (*p* < 0.05). UC: unchallenged control group, UT: unchallenged treatment group, CC: challenged control group, CT: challenged treatment group.

**Table 5:** Mean values obtained from real-time PCR and ELISA tests among the experimental groups at 23 and 29 days.

Day	Assay	Parameter	UC	UT	CC	CT
23 days	Real-Time PCR ^1^	MHC-I (lymph nodes)	13.27 ± 14.20	14.66 ± 14.20	38.22 ± 10.04	34.50 ± 10.04
		MHC-II (lymph nodes)	0.65 ± 0.17	1.40 ± 0.17	0.46 ± 0.12	0.74 ± 0.12
		IFN-γ (jejunum)	11.42 ± 45.39	7.71 ± 45.39	55.67 ± 32.10	3.98 ± 32.10
		IL-1β (jejunum)	0.57 ± 0.21	0.37 ± 0.21	0.57 ± 0.15	0.42 ± 0.15
		TLR2 (jejunum)	0.78 ± 3.28	1.37 ± 3.28	4.64 ± 2.32	0.93 ± 2.32
		TLR4 (jejunum)	1.09 ± 0.48	0.59 ± 0.48	0.96 ± 0.34	0.58 ± 0.34
	ELISA	IgA serum	0.57 ± 0.12	0.52 ± 0.12	0.43 ± 0.09	0.23 ± 0.09
		IgA scrape	98.93 ± 12.40	100.63 ± 12.40	113.30 ± 8.77	100.23 ± 8.77
		CXCL9 scrape	1.10 ± 0.78	2.42 ± 0.78	1.24 ± 0.55	1.85 ± 0.55
		TNF-α scrape	0.34 ± 0.39	1.34 ± 0.39	0.61 ± 0.27	0.89 ± 0.27
		IL-8 scrape	1.74 ± 1.29	3.84 ± 1.29	2.87 ± 0.91	2.44 ± 0.91
		IL-1 scrape	0.06 ± 0.05	0.18 ± 0.05	0.09 ± 0.03	0.10 ± 0.03
29 days	Real-Time PCR ^1^	MHC-I (lymph nodes)	12.31 ± 25.77	24.17 ± 25.77	71.11 ± 18.22	46.19 ± 18.22
		MHC-II (lymph nodes)	0.76 ± 0.30	0.86 ± 0.30	0.75 ± 0.21	0.82 ± 0.21
		IFN-γ (jejunum)	1.31 ± 7.95	21.56 ± 7.95	5.44 ± 5.62	1.74 ± 5.62
		IL-1β (jejunum)	0.55 ± 0.58	0.47 ± 0.58	0.62 ± 0.41	1.18 ± 0.41
		TLR2 (jejunum)	0.49 ± 6.80	18.16 ± 6.80	0.75 ± 4.81	0.66 ± 4.81
		TLR4 (jejunum)	0.43 ± 0.63	1.41 ± 0.63	0.64 ± 0.45	1.11 ± 0.45
	ELISA	IgA serum	0.25 ± 0.15	0.70 ± 0.15	0.26 ± 0.11	0.44 ± 0.10
		IgA scrape	105.96 ± 4.42	106.55 ± 4.42	99.34 ± 3.12	105.95 ± 3.12
		CXCL9 scrape	2.20 ± 0.55	3.14 ± 0.54	2.22 ± 0.39	2.07 ± 0.39
		TNF-α scrape	0.79 ± 0.38	1.65 ± 0.38	0.74 ± 0.27	0.82 ± 0.27
		IL_8 scrape	3.06 ± 0.71	4.40 ± 0.71	3.43 ± 0.50	3.34 ± 0.50
		IL-1 scrape	0.11 ± 0.04	0.14 ± 0.04	0.07 ± 0.03	0.11 ± 0.03

^1^ The data were normalized to beta-actin expression and reported as relative expression. TP: Total protein, UC: unchallenged control group, UT: unchallenged treatment group, CC: challenged control group, CT: challenged treatment group. The comparisons were performed using the Kruskal–Wallis test and the means were separated with the Wilcoxon rank sum test for comparing independent groups. The results are presented as least squared means ± standard error.

**Table 6:** Mean values obtained from real-time PCR and ELISA tests at 23 and 29 days divided by the experimental groups.

Group	Assay	Parameter	Day 23	Day 29
Unchallenged Control (UC)	Real-Time PCR ^1^	MHC-I (lymph nodes)	13.27 ± 10.64	12.31 ± 7.53
		MHC-II (lymph nodes)	0.65 ± 0.45	0.76 ± 0.32
		IFN-γ (jejunum)	11.42 ± 5.18	1.31 ± 3.66
		IL-1β (jejunum)	0.57 ± 0.25	0.55 ± 0.17
		TLR2 (jejunum)	0.78 ± 0.16	0.49 ± 0.11
		TLR4 (jejunum)	1.09 ± 0.37	0.43 ± 0.30
	ELISA	IgA serum	0.57 ± 0.06	0.25 ± 0.44
		IgA scrape	98.93 ± 4.16	105.96 ± 2.94
		CXCL9 scrape	1.10 ± 0.36	2.20 ± 0.26
		TNF-α scrape	0.34 ± 0.10	0.79 ± 0.07
		IL-8 scrape	1.74 ± 0.72	3.06 ± 0.51
		IL-1 scrape	0.06 ± 0.03	0.11 ± 0.02
Uchallenged Treatment (UT)	Real-Time PCR ^1^	MHC-I (lymph nodes)	14.66 ± 19.62	24.17 ± 13.17
		MHC-II (lymph nodes)	1.40 ± 0.53	0.86 ± 0.38
		IFN-γ (jejunum)	7.71 ± 22.25	21.56 ± 15.74
		IL-1β (jejunum)	0.37 ± 0.21	0.47 ± 0.15
		TLR2 (jejunum)	1.37 ± 21.48	18.16 ± 15.19
		TLR4 (jejunum)	0.59 ± 1.57	1.41 ± 0.82
	ELISA	IgA serum	0.52 ± 0.26	0.70 ± 0.19
		IgA scrape	100.63 ± 2.89	106.55 ± 2.04
		CXCL9 scrape	2.42 ± 0.72	3.14 ± 0.51
		TNF-α scrape	1.34 ± 1.02	1.65 ± 0.72
		IL-8 scrape	3.84 ± 1.10	4.40 ± 0.78
		IL-1 scrape	0.18 ± 0.07	0.14 ± 0.05
Challenged Control (CC)	Real-Time PCR ^1^	MHC-I (lymph nodes)	38.22 ± 30.11	71.11 ± 21.29
		MHC-II (lymph nodes)	0.46 ± 0.17	0.75 ± 0.12
		IFN-γ (jejunum)	55.67 ± 29.06	5.44 ± 20.55
		IL-1β (jejunum)	0.57 ± 0.17	0.62 ± 0.12
		TLR2 (jejunum)	4.64 ± 2.09	0.75 ± 1.48
		TLR4 (jejunum)	0.96 ± 0.32	0.64 ± 0.23
	ELISA	IgA serum	0.43 ± 0.10	0.26 ± 0.08
		IgA scrape	113.30 ± 7.88	99.34 ± 5.57
		CXCL9 scrape	1.24 ± 0.62	2.22 ± 0.44
		TNF-α scrape	0.61 ± 0.23	0.74 ± 0.16
		IL-8 scrape	2.87 ± 0.75	3.43 ± 0.53
		IL-1 scrape	0.09 ± 0.03	0.07 ± 0.02
Challegned Treatment (CT)	Real-Time PCR ^1^	MHC-I (lymph nodes)	34.50 ± 20.08	46.19 ± 14.20
		MHC-II (lymph nodes)	0.74 ± 0.25	0.82 ± 0.18
		IFN-γ (jejunum)	3.98 ± 1.32	1.74 ± 0.93
		IL-1β (jejunum)	0.42 ± 0.80	1.18 ± 0.56
		TLR2 (jejunum)	0.93 ± 2.29	0.66 ± 0.21
		TLR4 (jejunum)	0.58 ± 0.70	1.11 ± 0.50
	ELISA	IgA serum	0.23 ± 0.15	0.44 ± 0.11
		IgA scrape	100.23 ± 5.77	105.95 ± 4.08
		CXCL9 scrape	1.85 ± 0.58	2.07 ± 0.41
		TNF-α scrape	0.89 ± 0.28	0.82 ± 0.20
		IL-8 scrape	2.44 ± 0.88	3.34 ± 0.62
		IL-1 scrape	0.10 ± 0.05	0.11 ± 0.03

^1^ The data were normalized to beta-actin expression and reported as the relative expression. TP: Total protein, UC: unchallenged control group, UT: unchallenged treatment group, CC: challenged control group, CT: challenged treatment group. The comparisons were performed using the Wilcoxon rank sum test for the independent groups. The results are presented as least squared means ± standard error.

**Table 7:** Oligonucleotides used for PCR assays to detect engineered and wild-type tobacco seed lines.

Gene		Oligonucleotide Sequences	PCR Product Size (bp)
F18 adhesive fimbriae	313 F	5′ GGATCCATGAAAAGACTAGTGTTTATTTCTTTTGA	519
	314 R	3′ CGAATGCGCCAATGAATGTTCATTCTCGAG	
VT2e-B subunit	307 F	5′ GGATCCATGAAGAAGATGTTTATAGCGG	270
	308 R	3′ AACGGGTCCACTTCAAATTGATTCTCGAG	
NOS terminator	118 F	5′ GCATGACGTTATTTATGAGATGGG	118
	118 R	3′ GACACCGCGCGCGATAATTTATCC	

**Table 8:** Composition of experimental diets.

Ingredient, % as Fed	Basal Diet	High Protein Diet
Barley	22.80	16.19
Wheat flakes	16.80	11.93
Wheat meal	13.40	9.51
Maize flakes	11.20	7.95
Barley flakes	7.26	5.15
Soy protein concentrate	7.00	4.97
Soybean meal	-	2.80
Whey	5.56	3.95
Maize meal	5.20	3.69
Fish meal (herring)	3.97	2.82
Monohydrate dextrose	3.71	2.63
Spray-dried plasma	2.71	1.92
Coconut oil	2.70	1.92
Soybean oil	1.60	1.14
Dicalcium phosphate	0.52	0.37
Calcium carbonate	0.11	0.08
Sodium butyrate 30% ^1^	0.22	0.16
L-Lys	0.67	0.48
DL-Met	0.32	0.23
L-Thr	0.30	0.22
L-Trp	0.12	0.09
Vitamin/mineral premix ^2^	0.33	0.23
Vitamin E 50%	0.01	0.01
Additives: phytase ^3^, xylanase ^4^, Acidifiers ^5^, feed flavours	1.66	1.18
Calculated composition		
Dry matter	90.33	88.50
CP	17.80	25.42
EE	5.87	4.57
CF	2.19	3.20
Ash	4.46	5.17
Starch + sugar	51.51	38.88
Lysin	1.48	1.73
NE Mcal/kg	2.60	3.56

^1^ Palm oil, salts of fatty acids (sodium butyrate 30%), calcium carbonate. ^2^ Composition per kg of diet: Vitamin A, (E 672) 4,000,000 UI/kg; Vitamin D3, (E 671) 400,000 UI/kg; Vitamin E, (3a700) 40,000 mg/kg; Vitamin B1, 1200 mg/kg; Vitamin B2, 4000 mg/kg; Calcium D-pantothenate, (3a841) 10,865 mg/kg; Vitamin B6, (3a831) 2400 mg/kg; Vitamin B12, 16 mg/kg; niacinamide, (3a315) 14,000 mg/kg; Vitamin K3, 2000 mg/kg; Folic acid, (3a316) 600 mg/kg; D-Biotin, 80 mg/kg; Choline chloride, (3a890) 90,000 mg/kg, Fe (FeO), 45,000 mg/kg; Cu (CuSO_4_), 8000 mg/kg; Zn (ZnO), 52,200 mg/kg; Mn (MnO), 16,000 mg/kg; I (Ca(IO_3_)_2_), 240 mg/kg; Se (Na_2_SeO_3_), 120 mg/kg. ^3^ Phytase (EC 3.1.3.26) minimum: 10,000 Fytα-phytase/g. ^4^ Endo-1,4β-Xylanase (IUB/EC 3.2.1.8) minimum 1000 FXU/g. ^5^ Orthophosphoric acid 33.5%, Calcium formate 32.38%, Citric acid 7.8%, Fumaric Acid 5%, Silicic acid 6%.

**Table 9:** Oligonucleotides used for real-time PCR reactions.

Gene	Oligonucleotide Sequence (5′ to 3′)	Accession Number (GenBank)	PCR Product Size (bp)
TLR-2, F	GACACCGCCATCCTCATTCT	GU138028	130
TLR-2, R	CTTCCCGCTGCGTCTCAT		
TLR-4, F	GCCTTTCTCTCCTGCCTGAG	AB188301	83
TLR-4, R	AGCTCCATGCATTGGTAACTAATG		
IFN-γ, F	GCCAGGCGCCCTTTTTTA	NM_213948	121
IFN-γ, R	CTCTCCTCTTTCCAATTCTTCAAAAT		
IL1-β, F	ACGGTGACAACAATAATGACCTGT	NM_214055	81
IL1-β, R	CAAGGTCCAGGTTTTGGGTG		
MHC-I, F	CGCACAGACTTTCCGAGTG	AF464005	109
MHC-I, R	GTCTGGTCCCAAGTAGCAG		
MHC-II, F	CAAGCACTGGGAGTTTGAAG	DQ883222	125
MHC-II, R	ACACCCTTGATGATGAGGAC		
β-actin, F	CTCCTTCCTGGGCATGGAG	DQ452569	148
β-actin, R	GAGTTGAAGGTGGTCTCGTGG		

TLR, Toll-like receptor; IFN, interferon; IL, interleukin; MHC, major histocopmpatibility complex.

## Data Availability

All the data are available within the article and from the corresponding author upon reasonable request.

## References

[1] Szczepanik K., Furgał-Dierżuk I., Gala Ł., Świątkiewicz M. (2023). Effects of *Hermetia illucens* Larvae Meal and Astaxanthin as Feed Additives on Health and Production Indices in Weaned Pigs. Animals.

[2] Bonetti A., Tugnoli B., Piva A., Grilli E. (2021). Towards Zero Zinc Oxide: Feeding Strategies to Manage Post-Weaning Diarrhea in Piglets. Animals.

[3] Sun Y., Kim S. (2017). Intestinal challenge with enterotoxigenic *Escherichia coli* in pigs, and nutritional intervention to prevent postweaning diarrhea. Anim. Nutr..

[4] Vangroenweghe F.A. (2021). 238 Vaccination with an *E. coli* F4/F18 Vaccine for the Prevention of F4-ETEC Post-weaning Diarrhea Resulted in Reduced Post-weaning Mortality and Antibiotic Use. J. Anim. Sci..

[5] Dierick M., Ongena R., Vanrompay D., Devriendt B., Cox E. (2022). Lactoferrin Decreases Enterotoxigenic *Escherichia coli*-Induced Fluid Secretion and Bacterial Adhesion in the Porcine Small Intestine. Pharmaceutics.

[6] Shen J., Rump L., Ju W., Shao J., Zhao S., Brown E., Meng J. (2015). Virulence characterization of non-O157 Shiga toxin-producing *Escherichia coli* isolates from food, humans and animals. Food Microbiol..

[7] Tan C., Tang X., Zhang X., Ding Y., Zhao Z., Wu B., Cai X., Liu Z., He Q., Chen H. (2012). Serotypes and virulence genes of extraintestinal pathogenic *Escherichia coli* isolates from diseased pigs in China. Vet. J..

[8] Johannes L., Römer W. (2010). Shiga toxins—From cell biology to biomedical applications. Nat. Rev. Microbiol..

[9] Nainu F., Permana A.D., Djide N.J.N., Anjani Q.K., Utami R.N., Rumata N.R., Zhang J., Emran T.B., Simal-Gandara J. (2021). Pharmaceutical Approaches on Antimicrobial Resistance: Prospects and Challenges. Antibiotics.

[10] Cormican M., Hopkins S., Jarlier V., Reilly J., Simonsen G., Strauss R., Vandenberg O., Zabicka D., Zarb P., Catchpole M. (2017). ECDC, EFSA and EMA Joint Scientific Opinion on a list of outcome indicators as regards surveillance of antimicrobial resistance and antimicrobial consumption in humans and food-producing animals. Efsa J..

[11] Micoli F., Bagnoli F., Rappuoli R., Serruto D. (2021). The role of vaccines in combatting antimicrobial resistance. Nat. Rev. Microbiol..

[12] Rossi L., Dell’Orto V., Vagni S., Sala V., Reggi S., Baldi A. (2014). Protective effect of oral administration of transgenic tobacco seeds against verocytotoxic *Escherichia coli* strain in piglets. Vet. Res. Commun..

[13] Sohrab S.S. (2020). An edible vaccine development for coronavirus disease 2019: The concept. Clin. Exp. Vaccine Res..

[14] Rossi L., Pinotti L., Agazzi A., Dell’Orto V., Baldi A. (2014). Plant bioreactors for the antigenic hook-associated flgK protein expression. Ital. J. Anim. Sci..

[15] Rossi L., Turin L., Alborali G.L., Demartini E., Filipe J.F.S., Riva F., Riccaboni P., Scanziani E., Trevisi P., Dall’Ara P. (2021). Translational Approach to Induce and Evaluate Verocytotoxic, *E. coli* O138 Based Disease in Piglets. Animals.

[16] Lee S.-I., Ntakiyisumba E., Won G. (2022). Systematic review and network meta-analysis to compare vaccine effectiveness against porcine edema disease caused by Shiga toxin-producing *Escherichia coli*. Sci. Rep..

[17] Dubreuil J.D. (2021). Pig vaccination strategies based on enterotoxigenic *Escherichia coli* toxins. Braz. J. Microbiol..

[18] Vekemans J., Hasso-Agopsowicz M., Kang G., Hausdorff W.P., Fiore A., Tayler E., Klemm E.J., Laxminarayan R., Srikantiah P., Friede M. (2021). Leveraging vaccines to reduce antibiotic use and prevent antimicrobial resistance: A World Health Organization action framework. Clin. Infect. Dis..

[19] Coddens A., Loos M., Vanrompay D., Remon J.P., Cox E. (2017). Cranberry extract inhibits in vitro adhesion of F4 and F18+ *Escherichia coli* to pig intestinal epithelium and reduces in vivo excretion of pigs orally challenged with F18+ verotoxigenic *E. coli*. Vet. Microbiol..

[20] Cuccato M., Scaglione F.E., Centelleghe C., Divari S., Biolatti B., Pregel P., Cannizzo F.T. (2022). Assessment of Antimicrobial Effects on Broiler Gut Barrier through Histopathology and Immunohistochemistry of Tight-Junction Proteins. Front. Vet. Sci..

[21] Karttunen T.J., Turunen S. (2022). Lymphonodular Hyperplasia. Textb. Pediatr. Gastroenterol. Hepatol. Nutr. A Compr. Guide Pract..

[22] Shiu J., Piazuelo M.B., Ding H., Czinn S.J., Drakes M.L., Banerjee A., Basappa N., Kobayashi K.S., Fricke W.F., Blanchard T.G. (2015). Gastric LTi cells promote lymphoid follicle formation but are limited by IRAK-M and do not alter microbial growth. Mucosal Immunol..

[23] Li Y., Jin L., Chen T. (2020). The effects of secretory IgA in the mucosal immune system. BioMed Res. Int..

[24] Pasetti M.F., Simon J.K., Sztein M.B., Levine M.M. (2011). Immunology of gut mucosal vaccines. Immunol. Rev..

[25] Jabif M.F., Gumina E., Hall J.W., Hernandez-Velasco X., Layton S. (2021). Evaluation of a Novel Mucosal Administered Subunit Vaccine on Colostrum IgA and Serum IgG in Sows and Control of Enterotoxigenic *Escherichia coli* in Neonatal and Weanling Piglets: Proof of Concept. Front. Vet. Sci..

[26] Melkebeek V., Goddeeris B.M., Cox E. (2013). ETEC vaccination in pigs. Vet. Immunol. Immunopathol..

[27] Davitt C.J., Lavelle E.C. (2015). Delivery strategies to enhance oral vaccination against enteric infections. Adv. Drug Deliv. Rev..

[28] Hu C.X., Xu Y.X.Y., Hao H.N., Liu R.D., Jiang P., Long S.R., Wang Z.Q., Cui J. (2021). Oral vaccination with recombinant Lactobacillus plantarum encoding *Trichinella spiralis* inorganic pyrophosphatase elicited a protective immunity in BALB/c mice. PLoS Negl. Trop. Dis..

[29] Rossi L., Di Giancamillo A., Reggi S., Domeneghini C., Baldi A., Sala V., Dell’Orto V., Coddens A., Cox E., Fogher C. (2013). Expression of verocytotoxic *Escherichia coli* antigens in tobacco seeds and evaluation of gut immunity after oral administration in mouse model. J. Vet. Sci..

[30] Verdonck F., Cox E., van Gog K., Van der Stede Y., Duchateau L., Deprez P., Goddeeris B. (2002). Different kinetic of antibody responses following infection of newly weaned pigs with an F4 enterotoxigenic *Escherichia coli* strain or an F18 verotoxigenic *Escherichia coli* strain. Vaccine.

[31] Luise D., Lauridsen C., Bosi P., Trevisi P. (2019). Methodology and application of *Escherichia coli* F4 and F18 encoding infection models in post-weaning pigs. J. Anim. Sci. Biotechnol..

[32] NRC (2012). Nutrient Requirements of Swine.

[33] AOAC (2019). Official Methods of Analysis.

[34] AOCS, A.O.C. (2009). Crude Fiber Analysis in Feeds by Filter Bag Technique..

[35] Dell’Anno M., Callegari M.L., Reggi S., Caprarulo V., Giromini C., Spalletta A., Coranelli S., Rossi C.A.S., Rossi L. (2021). *Lactobacillus plantarum* and *Lactobacillus reuteri* as Functional Feed Additives to Prevent Diarrhoea in Weaned Piglets. Animals.

[36] Turin L., Tribbioli G., Invernizzi P., Grati F., Crema S., Laible G., Riva F. (2007). Fetal microchimerism in normal and embryo transfer bovine pregnancies. Vet. Res. Commun..

